# A multi-tissue gene expression dataset for hibernating brown bears

**DOI:** 10.1186/s12863-023-01136-3

**Published:** 2023-06-08

**Authors:** Blair W. Perry, Michael W. Saxton, Heiko T. Jansen, Corey R. Quackenbush, Brandon D. Evans Hutzenbiler, Charles T. Robbins, Joanna L. Kelley, Omar E. Cornejo

**Affiliations:** 1 School of Biological Sciences, Washington State University, Pullman, WA 99164 USA; 2 Department of Integrative Physiology and Neuroscience, Washington State University, Pullman, WA 99164 USA; 3 School of the Environment, Washington State University, Pullman, WA 99164 USA; 4 Department of Ecology and Evolutionary Biology, University of California, Santa Cruz, CA 95060 USA

**Keywords:** Gene expression, Hibernation, Transcriptomics, Brown bears

## Abstract

**Objectives:**

Complex physiological adaptations often involve the coordination of molecular responses across multiple tissues. Establishing transcriptomic resources for non-traditional model organisms with phenotypes of interest can provide a foundation for understanding the genomic basis of these phenotypes, and the degree to which these resemble, or contrast, those of traditional model organisms. Here, we present a one-of-a-kind gene expression dataset generated from multiple tissues of two hibernating brown bears (*Ursus arctos*).

**Data description:**

This dataset is comprised of 26 samples collected from 13 tissues of two hibernating brown bears. These samples were collected opportunistically and are typically not possible to attain, resulting in a highly unique and valuable gene expression dataset. In combination with previously published datasets, this new transcriptomic resource will facilitate detailed investigation of hibernation physiology in bears, and the potential to translate aspects of this biology to treat human disease.

## Objective

Multiple lineages of mammals, including ground squirrels [1–3], bears [4–6], and even several primates [7, 8], hibernate annually, during which metabolism is depressed, body temperature decreases to varying extents, and a suite of cellular and physiological changes culminate in the ability to survive periods of food scarcity [4, 5, 9–11]. Understanding how organisms both achieve and reverse hibernation phenotypes may provide translational insight into novel treatments for numerous human diseases, such as the reversal of insulin resistance to avoid subsequent onset of diabetes in humans [12, 13].

Studies of gene expression have proved valuable for identifying genes and signaling pathways underlying hibernation phenotypes in key tissues. For example, early microarray studies in bear heart, liver, and muscle revealed differential expression of genes associated with protein biosynthesis and lipid metabolism during hibernation [14–17], and subsequent studies utilizing mRNA-seq have since further characterized massive changes in gene expression that occur during hibernation in liver, muscle, and adipose tissue in bears [18–20]. These studies have focused on a small number of tissues per individual at a time, and our understanding of whole-body changes in gene expression associated with hibernation is therefore lacking and largely unexplored.

Here, we present an unprecedented transcriptomic dataset for 26 samples collected from 13 tissues of two hibernating brown bears (Table 1). These data were collected opportunistically rather than as part of a pre-designed experiment; as a result, this dataset is notably low in sample number (n = 2 bears) and does not include powerful controls (i.e., samples from all tissues in non-hibernating bears). However, these samples are exceedingly unique and are typically impossible to attain, and therefore present an inherently valuable and unprecedented look at gene expression across multiple tissues in hibernating bears.

**Table 1:** Overview of data files/data sets

Label	Name of data file/data set	File types (file extension)	Data repository and identifier (DOI or accession number)
Data file 1	Sample information table	MS Excel file (.xlsx)	Figshare (10.6084/m9.figshare.22574578.v1) [21]
Data file 2	Detailed methods information	MS Word file (.docx)	Figshare (10.6084/m9.figshare.22574578.v1) [21]
Data set 1	Multi-tissue transcriptomic dataset for hibernating brown bears	FASTQ files (.fastq)	NCBI Sequence Read Archive (https://identifiers.org/bioproject:PRJNA835146) [22]

## Data description

### Sample collection

Tissues used in this study were collected during the necropsy of two adult male bears (individuals P and R) following euthanization in winter 2016. Both bears were born in captivity in 2011 at the Washington State University Bear Center. The bears were anesthetized using a mixture of Tiletamine/Zolazepam (Telazol) and dexmedetomidine and subsequently euthanized in January 2016 with Sodium pentobarbital euthanasia solution, 1 ml/10 lbs administered intravenously. Procedures were conducted by staff of the Washington State University College of Veterinary Medicine. Samples were collected by the necropsy team immediately post-mortem to minimize nucleic acid degradation; samples were then placed in RNAlater (Invitrogen, Carlsbad, CA, USA) for storage. The following tissues were sampled: lung (right and left), heart ventricle (left and right), heart atrium (left and right), small intestine, kidney medulla, kidney cortex, gall bladder, adipose, stomach, gastrocnemius, liver, skin, and spleen. See Data file 1 [21] for additional sample information. Samples were transported to the laboratory where they were stored at -80° C. Procedures for all experiments were approved by the Institutional Animal Care and Use Committee at Washington State University (Protocol #06468).

### RNA extraction, library preparation, and sequencing

Tissue was homogenized using a TissueLyser LT (Qiagen, Redwood City, CA, USA) and RNA extractions were completed using the RNeasy fibrous tissue mini kit (Qiagen, Valencia, CA, USA) using a QIAcube (Qiagen). RNA yield and quality were assessed using a Qubit 2.0 (Invitrogen, Carlsbad, CA, USA) and Bioanalyzer 2100 (Agilent Technologies, Santa Clara, CA, USA), respectively. RNA-sequencing libraries were prepared using the Illumina TruSeq Stranded Total RNA Prep with Ribo-Zero Gold kit to remove rRNA according to the manufacturer’s instructions (Part #15,031,048 Rev.E, Illumina, San Diego, CA, USA). The libraries were sequenced on one lane of an Illumina HiSeq 2500 with v4 reagents with 100 basepair (bp) paired-end reads. Additional detailed methods are available in Data file 2 [21]. Raw sequence data are available at NCBI BioProject PRJNA835146 (https://identifiers.org/bioproject:PRJNA835146) [22].

## Limitations

- Small sample size (n = 2).
- Lack of experimental controls (i.e., samples from non-hibernating bears).

## Data Availability

The data described in this Data note can be freely and openly accessed on the NCBI Short Read Archive under BioProject: PRJNA835146 (https://identifiers.org/bioproject:PRJNA835146). Please see Table 1 and references [19–21] for details and links to the data.
